# Role of Plasma Membrane at Dielectric Relaxations and Intermembrane Interaction in Human Erythrocytes

**DOI:** 10.3390/membranes13070658

**Published:** 2023-07-11

**Authors:** Ivan T. Ivanov, Boyana K. Paarvanova

**Affiliations:** Department of Physics, Biophysics, Roentgenology and Radiology, Medical Faculty, Thracian University, 6000 Stara Zagora, Bulgaria; boiana_parvanovasz@abv.bg

**Keywords:** erythrocyte membrane, spectrin skeleton, erythrocyte aggregation, deformability, dielectric relaxation

## Abstract

Dielectric relaxations at 1.4 MHz (β_sp_) and 9 MHz (γ1_sp_) on the erythrocyte spectrin network were studied by dielectric spectroscopy using dense suspensions of erythrocytes and erythrocyte ghost membranes, subjected to extraction with up to 0.2% volume Triton-X-100. The step-wise extraction of up to 60% of membrane lipids preserved γ1_sp_ and gradually removed β_sp_-relaxation. On increasing the concentration up to 100 mM of NaCl at either side of erythrocyte plasma membranes, the β_sp_-relaxation was linearly enhanced, while the strength of γ1_sp_-relaxation remained unchanged. In media with NaCl between 100 and 150 mM β_sp_-relaxation became slightly inhibited, while γ1_sp_-relaxation almost disappeared, possibly due to the decreased electrostatic repulsion allowing erythrocytes to come into closer contact. When these media contained, at concentrations 10–30 mg/mL dextran (MW 7 kDa), polyethylene glycol or polyvinylpyrrolidone (40 kDa), or albumin or homologous plasma with equivalent concentration of albumin, the γ1_sp_-relaxation was about tenfold enhanced, while β_sp_-relaxation was strengthened or preserved. The results suggest the Maxwell–Vagner accumulation of ions on the lipid bilayer as an energy source for β_sp_-relaxation. While β_sp_-relaxation appears sensitive to erythrocyte membrane deformability, γ1_sp_-relaxation could be a sensitive marker for the inter-membrane interactions between erythrocytes.

## 1. Introduction

The unique mechanical properties of human erythrocytes are heavily influenced by the mechanical function of the erythrocyte plasma membrane. The latter consists of a lipid membrane (lipid bilayer with intercalated integral proteins, including the major proteins band 3 and glycophorin C) supported beneath by a network formed mainly by the third major membrane protein, the filamentous spectrin [1,2]. In native erythrocytes, about 95% of spectrin is in a tetrameric state and the rest of the spectrin is mainly dimeric [3].

In addition to the interaction of the spectrin network with the lipid bilayer, there are two protein bridges that attach the spectrin network to the lipid membrane: the band-3 tetramer–ankyrin–spectrin bridge and the glycophorin C-actin–spectrin bridge to which the band-3 dimer–adducin–spectrin bridge is associated [4]. Experimental as well as theoretical studies have indicated that the spectrin network endows the erythrocyte plasma membrane with shear elasticity, while the lipid bilayer is largely responsible for the bending rigidity of the plasma membrane [5,6,7,8,9].

Electric impedance in general and radiofrequency dielectric spectroscopy of cell suspensions in particular provide rich information on the structural and dielectric properties of cells [10]. Perhaps the most studied is the β-dispersion, an interfacial polarization due to charge accumulation on cellular membranes with a tail of γ-dispersion (molecular polarization due to contributions from other relaxation processes, namely, restricted motions of charges and dipoles associated to lipid and protein components of the plasma membrane), usually appearing between 100 kHz and 15 MHz [11,12,13,14,15,16,17]. Due to the high sensitivity of dielectric spectroscopy to interface changes and the molecular structure of cells and tissues [14,18], it could be helpful in studying the spectrin network of erythrocyte plasma membrane. Recently, a special approach of this method has been used at the temperature for heat denaturation of spectrin, *T*_A_ = 49.5 °C [19], to derive the admittance contribution, ΔY*(*f*) = Δ*Y*′(*f*) + *j.* Δ*Y*″(*f*), and capacitance contribution, ΔC*(*f*) = Δ*C*′(*f*) + *j.* Δ*C*_ds_″(*f*), of the spectrin network to the electric admittance, Y*(*f*), and capacitance, C*(*f*), of erythrocytes and erythrocyte ghost membranes [1,20]. Here, *j* is the imaginary unit (*j^2^* = −1), Δ*C*_ds_″(*f*) represented the curve of dielectric loss dissipated on the spectrin network, and *f* is the frequency.

The complex plain plot of admittance contribution, Δ*Y*″ vs. Δ*Y*′, showed two semicircles, revealing two single-time dielectric relaxations on the spectrin network centered at 1.4 MHz and 9 MHz, termed β_sp_- and γ1_sp_-relaxation, respectively. A model comprising two parallel circuits, each containing a resistance, R, and capacitance, C, connected in series, was used to express quantitatively the strengths of the two relaxations. For example, the strengths of the two relaxations markedly decreased under the impact of agents (glutaraldehyde, diamide, wheat germ agglutinin, metabolic starvation, hypertonic media) known to reduce erythrocyte membrane deformability and flicker (transversal oscillations of erythrocyte plasma membrane with the amplitude of up to 400 nm) [20]. 

The characteristic frequency, *f*_βsp_, of β_sp_-relaxation has been shown to be coupled to the characteristic frequency of the interface β-relaxation on the lipid membrane when the latter was shifted up and down by changing the concentration of NaCl in suspension medium. Below and around the *f*_βsp_, the electric field accumulates; through the access impedance, opposite ions on the outside and inner aspects of the lipid bilayer prevent the field from entering the cytosol [21]. Using low-polarizable electrodes of black platinum with area of 1 cm^2^, the left shoulder of β_sp_-relaxation was detected over the frequency interval of 100 kHz to 1 kHz (in fact to 75 Hz [22]) when, due to the interface polarization, the cytosol was effectively isolated from the outside field. Data have been presented evidencing that the strength of β_sp_-relaxation was decreased on disconnecting glycophorin C from the actin–spectrin junction [1]. Based on above the results, the β_sp_-relaxation was assumed to originate from a piezo effect, the transformation of mechanical deformation of spectrin filaments into dielectric polarization powered by the electrostriction of lipid membrane through the attachment sites of the spectrin network, predominantly the glycophorin C-spectrin–actin attachment site [1]. Similar electrostriction has been used to remove the organic solvent from the black lipid bilayer preparations [23]. Piezoelectricity is known for many biological macromolecules with asymmetric spiral shapes including collagen, which is similar to spectrin. The origin of piezoelectricity in biological macromolecules lies in the internal rotation of dipoles, and such dipoles are also present on spectrin (for a review of literature, see [24]).

The γ1_sp_-relaxation was detected at frequencies (3–15 MHz) that allowed the field to penetrate into cytosol. There, the field was assumed to go into resonance with the dipoles of spectrin [1]. The strength of this relaxation decreased after disconnection of the band-3 ankyrin–spectrin attachment site. The characteristic frequency, *f*_γ1sp_, of the γ1_sp_-relaxation was decreased by glycerol and increased at higher concentrations of ions in the cytosol of erythrocyte ghost membranes [24]; it was also increased in erythrocyte species with smaller size [25].

On the admittance contribution plot, Δ*Y*″ vs. Δ*Y*′, the β_sp_- and γ1_sp_-relaxations were expressed by comparable-by-size semicircles, the first one placed above and the second one below the real axis, indicating the positive and negative susceptance contributions of the spectrin network, Δ*Y*″(*f*), respectively [1]. To explain the opposite signs of these susceptance contributions, an electric equivalent circuit was proposed in which the resistance modeling the dielectric loss on spectrin during the γ1_sp_-relaxation was parallel to that of the active loss in cytosol, while the resistance modeling the dielectric loss on spectrin during the β_sp_-relaxation was in series with above-mentioned access impedance [25]. However, the dielectric loss curve of the spectrin network depicted a strong and positive bell-shaped curve over the frequencies of β_sp_-relaxation and no apparent loss over the frequencies of γ1_sp_-relaxation. This problem was now resolved by studying the two relaxations in erythrocyte ghost membranes subjected to mild extraction of membrane lipids (Triton shells) and in erythrocytes treated by acid medium (pH 5.2). 

The main goal of this work was to study the two relaxations when the erythrocytes were suspended at the volume fraction (hematocrit) close to the physiological one (45%) in isotonic media containing NaCl and albumin at concentrations similar to those in the blood plasma. While the γ1_sp_-relaxation remained unaffected, the β_sp_-relaxation was linearly enhanced on increasing the concentration of NaCl in the media up to 100 mM, rinsing either side of erythrocyte lipid membrane; however, it was totally eliminated on extraction of half the membrane lipids. These results underlined the important role of the lipid bilayer as an effective permeability barrier for ion accumulation supplying energy for β_sp_-relaxation at the expense of the outside field. A further increase in NaCl concentration in outer media to 150 mM weakened the relaxations, particularly the γ1_sp_-relaxation. The presence in outside media of albumin or autologous plasma with equivalent concentrations of albumin, as well as some membrane-inactive synthetic polymers with molecular weight of 7 to 40 kDa (polyethylene glycol, polyvinylpyrrolidone, dextran) at concentrations between 10 and 50 mg/mL, prevented the weakening of β_sp_-relaxation and strongly strengthened the γ1_sp_-relaxation. These results were interpreted in terms of electric double layer repulsion, which decreased in the high ionic media, allowing the cells to be brought closer to each other via their excluded volume attraction, thus inhibiting the relaxations, especially the γ1_sp_-relaxation. The presence of natural and synthetic polymers opposed these inter-membrane contacts, thus enhancing the relaxations. These results substantiate that dielectric relaxations in erythrocytes are physiologically important and possibly reflect some physiologically relevant properties of erythrocyte plasma membrane such as inter-membrane interaction (γ1_sp_-relaxation) and deformability, elasticity, and flicker (β_sp_-relaxation). In addition, the results underline the importance of albumin to distance the erythrocytes in the high ionic medium of blood, thus maintaining blood stability.

## 2. Materials and Methods

### 2.1. Materials

Bovine albumin, dextran, polyvinylpyrrolidone, polyethylene glycol, Triton-X-100 (polyethylene glycol p-(1,1,3,3-tetramethylbutyl)-phenyl ether), mannit, NaCl, KCl, MgCl_2_, and phosphate buffer were purchased from Sigma Chemicals Co., St. Louis, MO, USA. The citrate–phosphate (McIlvaine) buffer (0.15 M, pH 5.0) was prepared by dissolving 1.815 g of Na_2_HPO_4_·2H_2_O and 0.9605 g of citric acid to 100 mL dH_2_O and adjusting the pH of the obtained solution to pH 5.0 by addition of HCl or NaOH.

### 2.2. Isolation of Erythrocytes

Erythrocytes and their homologous plasma were isolated from the fresh blood samples of patients with healthy status in the University hospital of Thracian university, Stara Zagora, Bulgaria, according to the protocol N 10/5, June 2019, of ethnic commission of the Medical faculty, Thracian university, Stara Zagora, Bulgaria. Prior to use, the erythrocytes were washed once in isotonic (150 mM) NaCl saline and twice in a medium of 10 mM NaCl and 280 mM mannit. Except otherwise indicated, the centrifugations were conducted at 150× *g* for 7 min.

### 2.3. Isolation of Erythrocyte Ghost Membranes

A total of 1 mL of cold erythrocyte suspension, hematocrit 0.60, was vigorously diluted in 15 mL of 1 °C-cold hemolytic solution, containing 5 mM phosphate buffer, pH 7.8, and 2 mM MgCl_2_ and left in refrigerator for 5 min [26]. To obtain leaky membranes, the hemolysate was centrifuged (4000× *g*, 12 min) and the membranes were isolated and once washed in the same hemolytic solution prior to usage. To obtain resealed membranes, the hemolysate was diluted with a cold solution containing NaCl and mannit to the indicated final concentrations and resealed (37 °C, 20 min). The resealed membranes were isolated (4000× *g*, 12 min) and washed in excess volume of the final testing media. 

### 2.4. Preparation of Triton-X-100 Shells of Erythrocyte Ghost Membranes

One volume of cold leaky membranes was vigorously mixed with equal volume of 1 °C-cold lipid-extraction medium containing 5 mM phosphate buffer, pH 7.8, 4 mM MgCl_2_, Triton-X-100 (up to 0.4% *v*/*v*), and NaCl (between 10 and 150 mM, as indicated). Because the Triton-X-100 is a chelator of Mg^2+^, these ions were used at higher concentration (4 mM). On contact of membranes with Triton-X-100, the opaque suspension became transparent. The extraction of membrane lipids lasted 30 min at 4 °C. The obtained dispersion was diluted by 3 volumes of washing medium of 5 mM phosphate buffer, pH 7.8, 4 mM MgCl_2_, and NaCl (between 10 and 150 mM, as indicated). Next, the mixture was centrifuged (8000× *g*, 12 min) to isolate the Trition shells [27]. The lowest pink button was discarded, and the overlying white layer of packed Triton shells was isolated and washed of Triton-X-100 three times in excess volume of the same cold washing medium prior to usage. 

The residual protease activity, originating from the white blood cell contamination of isolated erythrocytes, could damage the proteins of Triton shells [28]. To reduce it at the stage of erythrocyte isolation, the buffy coat, containing the white blood cells, was carefully discarded and the erythrocytes were washed three folds. The usage of erythrocyte ghost membranes instead of whole erythrocytes to produce Triton shells additionally reduced the protease activity.

### 2.5. Dielectric Spectroscopy of the Suspensions of Erythrocytes, Erythrocyte Ghost Membranes, and Triton Shells

Prior to testing the erythrocytes and resealed erythrocyte ghost membranes, they were suspended in the indicated isotonic solution, hematocrit 45%. A total of 70 μL of suspension or packed Triton shells was introduced with a syringe into the working conductometric cuvette (conductometric constant, K = 6.5 cm^−1^) which, in turn, was inserted tightly into a hole within an aluminum thermal block. After thermal equilibration, the block was heated at a heating rate of 1.5 °C/min. During heating, the complex admittance, Y* = *Y*′ + *j. Y*″, and complex capacitance, C* = *C*′ − *j. C*″, of tested sample were continuously measured and separated into their real (*Y*′, *C*′) and imaginary (*Y*″, *C*″) parts using an Impedance analyzer (currently available as Solartron 1260A Frequency Response Analyzer, Ametek Scientific Instruments, England), controlled by a computer. The values of Y* and C* were scanned at 16 frequencies between 30 kHz and 15 MHz with an integration time of 1 s. The cross-section of the aluminum thermal block was shown previously [20]. The applied inter-electrode voltage of 100 mV created a field far weaker than those used for the dielectrophoresis and electrophoresis of erythrocytes [29].

### 2.6. Dielectric Loss Curve of Triton Shells and the Suspensions of Erythrocytes and Erythrocyte Ghost Membranes 

**Figure 1:**
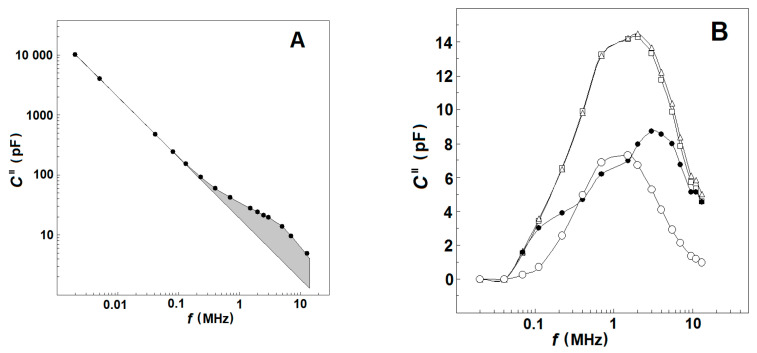
(**A**) Effect of frequency on the electric energy dissipated in suspensions of erythrocytes and erythrocyte ghost membranes. The energy loss (imaginary capacitance, *C*″/pF) of suspension is plotted against the frequency of electric field, *f*/MHz. The hematocrit and temperature were 45% and 25 °C, respectively. The straight line represents the conduction loss in the suspension while the shadowed area corresponds to the dielectric loss curve, Δ*C*_d_″/pF, of plasma membranes. (**B**) Impact of temperature on the frequency curve, Δ*C*_d_″/pF vs. *f*/MHz, of dielectric loss dissipated in the suspension. The temperature of the suspension was 41 °C (☐), 47 °C (Δ) and 53 °C (●). The temperature-corrected differential dielectric loss curve at *T*_A_, Δ*C*_ds_″/pF, is indicated by (○). For this and the next figures, the number of experiments was at least three, shown is the typical one.

At a given frequency, *f*, and temperature the dissipation, *C*″, of the tested sample represents the rate at which the electric field energy is dissipated due to the conduction of ions and oscillation of electric dipoles [30]. The conduction loss decreases with the reciprocal of *f* and, consequently, strongly prevails at low frequencies. At the radio frequency range, it becomes smaller than the dielectric loss as the latter peaks at the relaxation frequency of dipoles (Figure 1A). This type of frequency dependence of log (*C*″) allows us to discriminate both types of energy loss on the frequency domain. At frequencies between 10 Hz and 0.1 MHz, the dielectric loss of human blood is negligible with respect to conduction loss, and the log (*C*″) linearly declines with log (*f*) [30]. Above 0.1 MHz, the *log* (*C*″)/*log* (*f*) dependence is no longer linear because it mainly represents the energy loss due to dipole relaxations. 

Based on the above, the values of *C*″(*f*), measured within the frequency interval of 30–50 kHz, were used to obtain an analytical (e.g., power law) expression for the conduction loss valid both at low and high frequencies. The conduction loss data, calculated from this expression for the radio-frequency range of 0.1–15 MHz, were subtracted from the experimentally measured values of *C*″(*f*), and the remainder was defined as the dielectric loss curve, *C*_d_″(*f*), of tested suspension at the chosen temperature (exemplified by Figure 1B, open squares and triangles) [25]. 

### 2.7. Contribution of the Spectrin Network to the Dielectric Properties of Erythrocytes, Erythrocyte Ghost Membrane, and Triton Shells

Lipid membrane and its small width (≈7 nm) chiefly determine the dielectric properties (conductance, capacitance) of erythrocyte plasma membrane. The idea to take into account the spectrin network contribution originates from the conception that the negatively charged network of elastic spectrin filaments is well separated from the lipid bilayer and exercises its impact mainly through the long (≈30 nm) attachment bridges and by its electrostatic repulsion [31] from the negatively charged cytoplasmic aspect of the lipid bilayer. In addition to its physical separation, the spectrin network demonstrates distinct thermal properties—low temperature of denaturation, *T*_A_ (49.5 °C), and high activation energy of heat denaturation [19]. Thus, the dielectric contribution of the spectrin network could be specifically eliminated by rapid heating across *T*_A_, leaving the lipid membrane in a relatively intact state within the short time interval (≈1 min) of dielectric measurement. 

The heat-induced elimination of the dielectric contribution of the spectrin network has been demonstrated by the huge sigmoid and frequency-dependent changes in *Y*′, *Y*″, *C*′, and *C*″ taking place within the temperature interval of about 6 °C centered at *T*_A_ [24]. Each dielectric change was defined as the value at the native state minus the value at the denatured state of spectrin, respectively. However, in addition to the main component, induced by the spectrin denaturation, each change contained a minor component due to the effect of temperature on processes involving the cytosol and suspension medium. Compared to the former, the latter component was small and linear because it was not caused by protein denaturation and arose within a narrow temperature interval. Hence, it was determined for an equal temperature interval prior to spectrin denaturation and subtracted from the registered changes in *Y*′, *Y*″, and *C*′ at *T*_A_ as described previously [20,25]. This temperature correction extracted the spectrin-linked portion of the dielectric changes at T_A_ (the dielectric contribution of spectrin).

At a given temperature, the dielectric loss of the tested sample is represented by the area under the dielectric loss curve, *C*_d_″(*f*) (Figure 1B, open squares and triangles). This area is proportional to the total amount of dipoles, irrespective of their dipole moments and spatial distribution [32]. The dielectric loss curve, *C*_d_″(*f*), practically did not change its frequency profile within the temperature intervals of 30–47 °C and 53–59 °C. However, it abruptly shrunk at *T*_A_, indicating substantial reduction of the dielectric loss as the dipoles of denatured spectrin ceased to contribute. To obtain the dielectric loss curve of the spectrin network, ∆*C*_ds_″(*f*), the dielectric loss curve of the tested sample at 53 °C was subtracted from that at 47 °C and the obtained bell-shaped curve (Figure 1B, open circles) was corrected by temperature using the data for dielectric loss curves at 41 °C and 47 °C [25]. 

At each frequency the extracted, spectrin-linked contributions to the dielectric properties of the tested sample just prior to *T*_A_ are further noted as Δ*Y*′(*f*), Δ*Y*″(*f*), Δ*C*′(*f*), and Δ*C*_ds_″(*f*). 

### 2.8. Reduction of Electrode Polarization and Measurement Errors

With a two-electrode system submerged in a sample cell suspension, a counterion layer forms at each electrode whose potential drop reduces the electric field available to drive charges and rotate dipoles in the suspension [33]. This results in the apparently low conductivity and higher capacity of suspension. The effect increases with increasing sample conductivity and decreases with the increasing frequency and volume fraction of cells in the suspension, and its consequences are more pronounced on the capacitance than the conductance of suspension. For cell suspensions with low volume fractions (6%) and highly conductive medium (150 mM NaCl), the electrode polarization is important up to 200 kHz [34]. In our study, it was reduced using dense suspensions (hematocrit values about 45% close to that in blood), suspension media with low conductivity (isotonic solution of 10 mM NaCl and 280 mM mannit), and low polarizable platinum and Ag/AgCl electrodes. Instead of the absolute value of a chosen dielectric parameter, obtained at a given temperature and frequency, we used the difference between two different values of the parameter, one obtained at a temperature prior to the heat denaturation of spectrin and the other after. The two temperatures were close enough to affect the electrode polarization similarly; in addition, the temperature course of electrode polarization was corrected as explained in Section 2.6. Therefore, the spectrin denaturation strongly affected the difference between the two chosen values of the parameter while the impact of electrode polarization was strongly reduced. These measures made the effect of electrode polarization insignificant for frequencies above 40 kHz. Increasing the inter-electrode distance from 3 to 10 mm reduced by about three times the suspension static capacitance, preserving the capacitance due to electrode polarization. The comparison of the low-frequency spectrum of erythrocyte suspension capacitance at the two different inter-electrode distances confirmed the conclusion that the electrode polarization was not important above 40 kHz.

Except the errors due to electrode polarization, the usage of differentials (Δ*Y*′, Δ*Y*″, Δ*C*′, Δ*C*″) instead of the dielectric parameters (*Y*′, *Y*″, *C*′, *C*″) strongly decreased the errors due to the penetration of the current into cell cytosol, ion conductance, effect of temperature on the mobility of ions, and stray currents. The applied temperature correction procedure (Section 2.6) additionally decreased measurement errors. The most important source of deviation in the results was the origin of tested erythrocytes (the blood donor) and the metabolic exhaustion of erythrocytes. Therefore, all trials of each experiment were conducted with erythrocytes obtained from the same blood sample, as fresh as possible, within the time frame of about 5 to 6 h, during which the washed erythrocytes were kept as a dense paste in refrigerator. At such conditions, the reproducibility in the strength of relaxations was within the frames of ±3 to 4% relative to the mean values given in the tables. For a result to be reported, it should be reproduced with erythrocytes from at least three different blood samples.

## 3. Results

**Figure 2:**
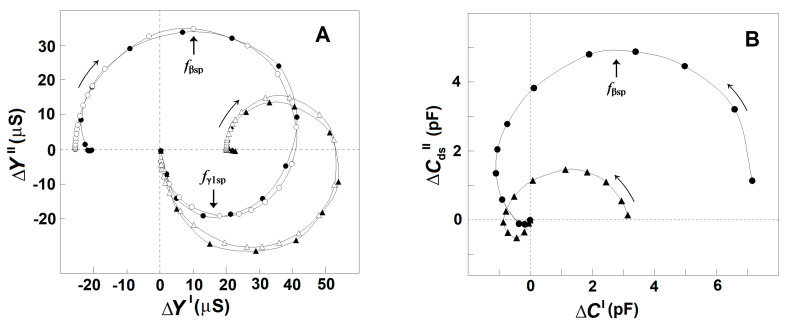
Effect of the acid treatment of erythrocytes on the admittance contribution plot, Δ*Y*″ vs. Δ*Y*′ (**A**), and capacitance contribution plot, Δ*C*_ds_″ vs. Δ*C*′ (**B**), of the spectrin network. Δ*Y*″/μS is presented as s function of Δ*Y*′/μS (**A**), while Δ*C*_ds_″/pF is presented as s function of Δ*C*′/pF (**B**). The erythrocytes were treated in 130 mM NaCl saline, containing 25 mM citrate/HCl buffer with pH 7.4 (●) or pH 5.2 (▲), hematocrit 10%, at 4 °C for 90 min. The suspension of treated erythrocytes was centrifuged, and the isolated paste of packed cells was tested. The open symbols on the plot of admittance contribution (**A**) indicate the model fit compared to the experimental data (full symbols). In this and the next figures, the curved arrows indicate the increase in frequency, while the straight line arrows indicate the characteristic frequencies of dielectric relaxations.

Based on the dielectroscopic methods for data processing [35,36], the implicit frequency dependence of the dielectric contribution of the spectrin network to the dielectric properties of the tested membranes was studied using the complex plane plots of ΔY″ vs. ΔY′ (Figure 2A, full circles) and ΔC_ds_″ vs. ΔC′ (Figure 2B, full circles). The two perfect semicircles on the admittance contribution plot, ΔY″ vs. ΔY′, (Figure 2A, full circles) have been attributed to two single-time dielectric relaxations on the spectrin network, marked as β_sp_ and γ1_sp_, respectively [20]. The (positive) semicircle above the real axis revealed the β_sp_-relaxation, while the (negative) semicircle below the real axis expressed the γ1_sp_ relaxation. On the capacitance contribution plot, ΔC_ds_″ vs. ΔC′, (Figure 2B, full circles) the β_sp_-relaxation was revealed by the semicircle placed predominantly above the real axis [1], while the γ1_sp_-relaxation possibly corresponded to the small offset attached at the high-frequency end. The characteristic frequencies, f_βsp_ and f_γ1sp_, of the β_sp_- and γ1_sp_-relaxations, respectively, are indicated by the arrows in Figure 2A as explained earlier [25].

### 3.1. Model Presentation of the Dielectric Relaxations on the Spectrin Network of Erythrocytes and Erythrocyte Ghost Membranes

In this report, the β_sp_- and γ1_sp_-relaxations on the Δ*Y*″ vs. Δ*Y*′ plot were modeled by an appropriate equivalent electric circuit as described previously [20,25]. The model presentation was used to better differentiate the two relaxations and describe them by quantitative parameters. The basic part of the model contained a resistor, *R*, and capacitor, *C*, connected in series, whose admittance plot represents a semicircle used to illustrate a single-time dielectric relaxation. The model consisted of two such circuits, connected in parallel, each one representing a different relaxation. The model parameters, *C*_βsp_ and *R*_βsp_ = 1/*Y*_βsp_, of the first circuit were the best fit values for the β_sp_-relaxation, while the *C*_γ1sp_ and *R*_γ1sp_ = 1/*Y*_γ1sp_ of the second circuit represented the best fit values for the γ1_sp_ relaxation. Using the measured characteristic frequencies of the two relaxations, *f*_βsp_ and *f*_γ1sp_, and two experimental values for the real admittance, the initial approximate values of all resistive and capacitive elements were calculated. After several iteration steps, they were adjusted, resulting in good enough fit between the admittance plot of the model circuit and the experimentally obtained admittance plot of the tested sample as shown in Figure 2A (open circles). The strengths of β_sp_- and γ1_sp_-relaxation, *Y*_βsp_, and *Y*_γ1sp_, respectively, were expressed by the apparent radiuses of their respective semicircles in Figure 2A and are shown in Table 1. 

In general, the capacitance is a receptacle of charges, i.e., electric energy, and the electric resistance is a dissipater of electric energy. Hence, the *R*_γ1sp_ and *R*_βsp_ could indicate the rate of energy dissipation on the spectrin network during the γ1_sp_- and β_sp_-relaxations, respectively. On the other hand, the *C*_γ1sp_ and *C_βsp_* could indicate the charge (energy), reversibly deposited in a half period on the spectrin network during the γ1_sp_- and β_sp_-relaxations, respectively. Since these parameters depend on the density (hematocrit) of the tested sample, it is more convenient to use their ratios. Such are the ratio, −*Y*_βsp_/*Y*_γ1sp_, of the energy dissipation rate and the charge accumulation ratio, −*C*_βsp_/*C*_γ1sp_, representing the dissipated energy rate and stored charges, respectively, on the spectrin network during the β_sp_-relaxation relative to that during the γ1_sp_-relaxation. In previous studies [1,37], the −*Y*_βsp_/*Y*_γ1sp_ ratio (~1.20 in control erythrocytes) has been shown to increase on the severing of the band-3 ankyrin–spectrin bridge in the presence of aprotic solvents, while it decreased on the severing of the glycophorin C-actin–spectrin bridge in the presence of protic solvents. In control erythrocytes, the −*C*_βsp_/*C*_γ1sp_ has too large a value (~7.70), in line with the high efficiency of the piezo effect for charge generation during the β_sp_-relaxation [25]. 

### 3.2. Complex Admittance and Capacitance Contribution of Spectrin Network as Affected by the Modification of Lipid Membrane

#### 3.2.1. Effect of Acidification of Erythrocytes on the Spectrin Relaxations

A previous study [1] has shown that DNAse I (Deoxyribonuclease I), known to depolymerize fibrillar actin [38]; diphosphoglycerate, known to weaken specifically the spectrin–actin interactions [39]; and urea, which, at the concentrations of 1–3 M, induces pre-denaturation perturbance in the structure of actin [40] and spectrin [41], all specifically inhibited the β_sp_ relaxation in erythrocyte and erythrocyte ghost membranes. In this study, the acidic medium (pH 5.2), which is known to acidify the cytosol of erythrocytes and detach the glycophorin C integral protein from the actin–spectrin junction of the membrane skeleton [42], also inhibited, strongly and specifically, the β_sp_ relaxation in erythrocyte, as explained below. 

The ΔY″ vs. ΔY′ plot (Figure 2A) and Table 1 demonstrate that the strength of β_sp_-relaxation was markedly reduced in erythrocytes incubated in acidic medium compared to erythrocytes incubated in medium with neutral pH at the same temperature and for the same time intervals. The acidic reaction of suspension medium caused the acidification of cytosol [43] and the detachment of about half the glycophorin C copies from the spectrin network [42]. The inhibitory effect of acidic pH on the β_sp_-relaxation is also demonstrated by the decrease in the radius and area of the large positive semicircle in the ΔC_ds_″ vs. ΔC′ plot (Figure 2B), which has been shown to reflect the β_sp_-relaxation in erythrocytes [1].

Comparing acid-treated erythrocytes to erythrocytes treated by medium with neutral pH, it is evident that in contrast to the inhibition of β_sp_-relaxation, the γ1_sp_-relaxation was apparently enhanced in acid-treated erythrocytes. This is shown by the enlargement of the semicircle placed below the real axis of the ΔY″ vs. ΔY′ plot (Figure 2A) and the appearance of a small negative semicircle attached at the high-frequency end of the ΔC_ds_″ vs. ΔC′ plot of acidified erythrocytes (Figure 2B).

#### 3.2.2. Effect of Mild Delipidation of Erythrocyte Ghost Membranes on the Spectrin Relaxations

**Figure 3:**
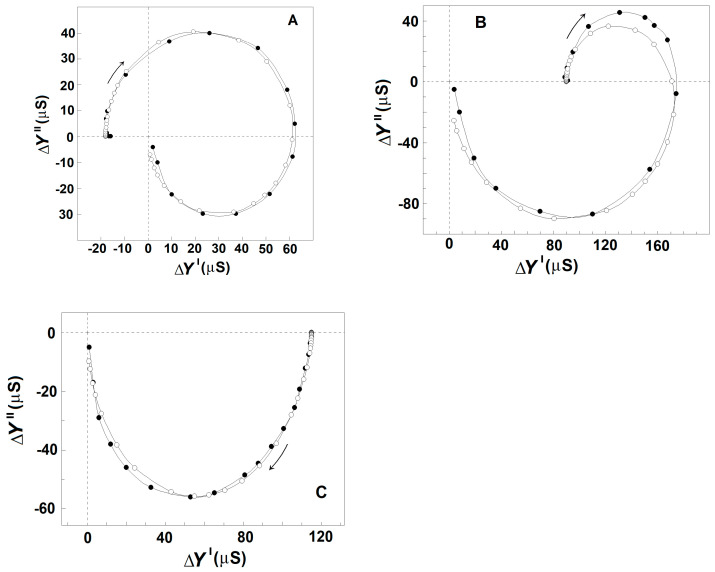
Effect of mild delipidation of erythrocyte ghost membranes on the admittance contribution plot ΔY″ vs. ΔY′ of the spectrin network. ΔY″/μS is presented as a function of ΔY′/μS. (**A**) Erythrocyte ghost membranes, resealed with 75 mM NaCl, 5 mM phosphate buffer, pH 7.8, and 2 mM MgSO_4_, were suspended in 5 mM NaCl and 140 mM mannit, hematocrit 0.45, and tested. To prepare Triton shells, the erythrocyte ghost membranes were suspended at hematocrit 0.30 in the lipid-extraction medium of 10 mM NaCl, 5 mM phosphate buffer, pH 7.8, 4 mM MgSO_4_, and Triton-X-100 at concentrations of 0.07 (**B**) and 0.20 (**C**) volume %, temperature 4 °C, for 90 min. The obtained Triton shells were isolated (6000× *g*, 12 min) and washed thrice of the detergent, and a packed sample of them was tested. The open symbols (○) indicate the model fit compared to the experimental data (●).

Figure 3A shows the admittance contribution plot of spectrin network in resealed erythrocyte ghost membranes. This plot is almost identical to the plot obtained with native erythrocytes (Figure 2A), indicating that compared to native erythrocytes, similar β_sp_- and γ1_sp_-relaxations took place on the spectrin network of erythrocyte ghost membranes. Next, the relaxations in erythrocyte ghost membranes were compared to those in Triton shells produced by the delipidation of erythrocyte ghost membranes by Triton-X-100 at concentrations up to 0.2% (*v*/*v*) (3.40 mM) (Figure 3B,C and Table 2). According to [27], the remaining lipids in Triton shells, produced by extraction with 0.1 and 0.2% (*v*/*v*) Triton-X-100, amounted to about 50% and 40%, respectively.

Figure 3 shows that in contrast to γ1_sp_-relaxation, the β_sp_-relaxation was as strongly inhibited as was the degree of lipid extraction. The strength of β_sp_-relaxation was maximal in erythrocyte ghost membranes, having an intact lipid bilayer, and it progressively diminished to zero, increasing the concentration of the detergent up to 0.1 volume % (1.70 mM). According to the recent study of Habibi et al. [44], this concentration of the detergent is about five times its critical concentration of micelle formation (0.22 mM) and about three times its concentration, inducing 100% hemolysis of erythrocytes (0.5 mM). 

Based on the data of [27], it could be assumed that the specific inhibition of β_sp_-relaxation, obtained after the treatment of erythrocyte ghost membranes with Triton-X-100, was caused by the extraction of about half of the membrane lipids, predominantly the glycolipids, and of significant portion of glycoproteins (predominantly the glycophorin C), while a substantial portion of the band 3 remained in Triton shells. The differential scanning calorimetry study [45] has also indicated that alongside the peripheral protein spectrin, a significant portion of band-3 integral protein remained in Triton shells prepared at such conditions.

#### 3.2.3. Effects of NaCl Concentration on the Strength, Y_γ1sp_, and Characteristic Frequency, f_γ1sp_, of γ1_sp_-Relaxation in Moderately Delipidated Triton Shells

**Figure 4:**
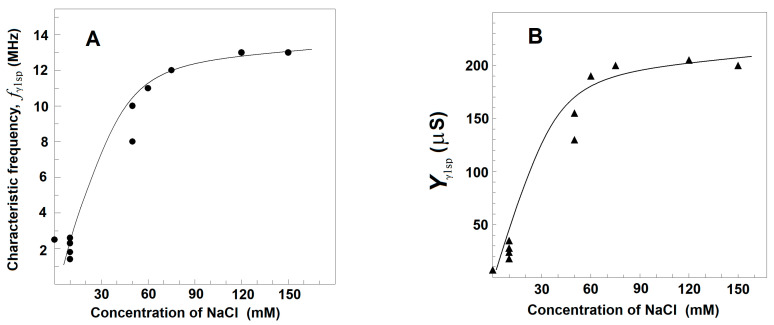
Effect of the concentration of NaCl in testing media on the characteristic frequency, f_γ1sp_, (**A**) and strength, Y_γ1sp_, (**B**) of γ1_sp_-relaxation in Triton shells of erythrocyte ghost membranes. f_γ1sp_/MHz (**A**) and Y_γ1sp_/μS (**B**) are presented as functions of NaCl concentration/mM. The Triton shells were prepared as explained for Figure 3C and thrice washed in a medium of 5 mM phosphate buffer, pH 7.4, 4 mM MgSO_4_, and the indicated concentration of NaCl. The obtained Triton shells were isolated (6000× *g*, 12 min) and a 70 μL packed sample of them was tested. The experimental data points (●, ▲) are modeled by the presented trend lines.

Using the plot of spectrin′s admittance contribution, the γ1_sp_-relaxation was studied in Triton shells produced at a certain concentration of the detergent (0.1% (*v*/*v*) that totally removed the β_sp_-relaxation. The final conclusion was that both the f_γ1sp_ and Y_γ1sp_ did not depend on the concentration of NaCl in the initial medium, used to extract the lipids. However, they were markedly sensitive to the NaCl concentration in the medium, used to wash the obtained shells from the detergent, i.e., in the final testing medium. At the same concentration of NaCl in the washing medium, e.g., 10 mM, f_γ1sp_, and Y_γ1sp_ did not vary when the concentration of NaCl in the medium, used to extract the lipids from Triton shells, varied between 10 and 150 mM (not shown). However, whatever the NaCl concentration in the extracting medium was, the f_γ1sp_ and Y_γ1sp_ increased, reaching saturation upon increasing the NaCl concentration of washing medium from 0 to 150 mM (Figure 4). At low concentration of NaCl (≤10 mM), the f_γ1sp_ was about 0.8–1.5 MHz; while at 150 mM, NaCl it reached the saturation level of about 13 MHz (Figure 4A). Similar dependence on the NaCl concentration was obtained for Y_γ1sp_ (Figure 4B). 

The results, shown in Figure 4, could be explained by the notion that the resting volume of the spectrin network, and therefore of Triton shell, depended on the equilibrium between the elastic force, which shrinks the spectrin network, and the electrostatic repulsion force, which expands the spectrin network. At higher concentrations of NaCl, the resulted screening of negative charges, predominating on spectrin filaments at neutral pH, weakened the repulsion force, resulting in the elastic shrinkage to a new equilibrium point of erythrocyte ghost membranes [46] and Triton shells [46,47]. Hence, the increase in the strength of γ1_sp_-relaxation at higher NaCl concentrations (Figure 4B) could be due to the larger number of Triton shells packed in the tested sample. The accompanying increase in the frequency, f_γ1sp_, of the γ1_sp_-relaxation at higher NaCl concentration (Figure 4A) has been earlier explained, modelling the spectrin filament by a hanging loaded spring [24]. The length of the spring expands by the weight of a hanging body (the electrostatic repulsion). At higher NaCl concentrations, the weight of the hanging body has to be decreased, causing a decrease in the length of the spring; hence, the frequency of its natural vibrations, i.e., the f_γ1sp_, should increase.

#### 3.2.4. Complex Capacitance Contribution of the Spectrin Network, ΔC′ vs. f and ΔC_ds_″ vs. f in Erythrocytes, Erythrocyte Ghost Membranes, and Triton Shells

**Figure 5:**
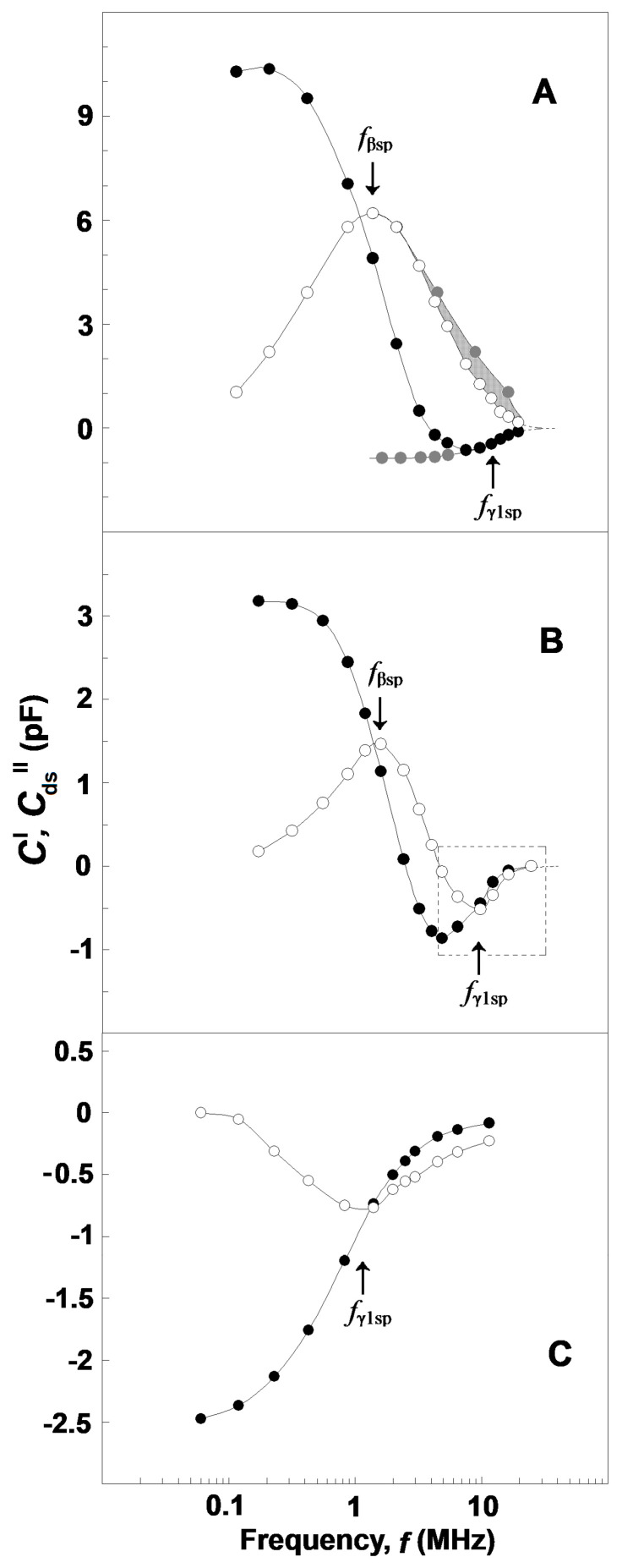
Frequency dependence of the complex capacitance contribution, ΔC′ vs. f (●) and ΔC_ds_″ vs. f (○), for erythrocytes and erythrocyte ghost membranes (**A**) and Triton shells (**B**,**C**). ΔC′/pF and ΔC_ds_″/pF are presented as a function of f/MHz. The erythrocytes were suspended in isotonic 10 mM NaCl/mannit medium, while erythrocyte ghost membranes (resealed with 75 mM NaCl, 5 mM phosphate buffer, pH 7.8 and 2 mM MgSO_4_) were suspended in 5 mM NaCl and 140 mM mannit, both at hematocrit 45%, and tested. The Triton shells were prepared as explained for Figure 3C at the following conditions: (**B**) extraction of the erythrocyte ghost membrane lipids with 0.07% Triton-X-100 and washing and packing of residues in 50 mM NaCl; (**C**) extraction of erythrocyte ghost membranes with 0.20% Triton-X-100 and washing and packing of residues in 10 mM NaCl. The obtained Triton shells were isolated (6000× *g*, 12 min) and 70 μL packed paste of them was tested. The inflection points on the ΔC′ vs. f curves, i.e., the top peak points of the ΔC_ds_″ vs. f curves, correspond to the characteristic frequencies, f_βsp_ and f_γ1sp_, as pointed out by the arrows. Other details are contained in the main text.

The two relaxations on the spectrin network of native erythrocytes and their ghost membranes were displayed by the capacitance contribution curve, ΔC′ vs. f (Figure 5A, full circles), as two sigmoid changes; a huge drop at f_βsp_ (≈1.4 MHz), depicting the β_sp_-relaxation; and a small rise at f_γ1sp_ (≈9 MHz), reflecting the γ1_sp_-relaxation [1]. Note the opposite directions of the frequency-induced changes in ΔC′ during the two relaxations. The dielectric loss curve, ΔC_ds_″ vs. f (Figure 5A, open circles), clearly exhibited only one huge bell-shaped positive peak at f_βsp_, while the expected negative peak centered at f_γ1sp_ was apparently absent. 

Compared to the ΔC′ vs. f curve of whole erythrocytes and their ghost membranes (Figure 5A, full circles), similar threshold changes were registered on the ΔC′ vs. f curve of Triton shells prepared by mild delipidation of erythrocyte ghost membranes with 0.07 volume % Triton-X-100 (Figure 5B, full circles) and of erythrocytes treated by acid medium (pH 5.2) (not shown). In these cases, the drop of ΔC′ at f_βsp_ was about threefold reduced by magnitude, while the rise at f_γ1sp_ was apparently enforced. Relatedly, the dielectric loss curve, ΔC_ds_″ vs. f, of Triton shells (Figure 5B, open circles) and acid-treated erythrocytes peaked two times at the two inflection points on the ΔC′ vs. f curve, which corresponded to the drop and rise of ΔC′. The first peak was positive and centered at f_βsp_, thus depicting the dielectric loss curve of β_sp_-relaxation in line with the data for erythrocytes and erythrocyte ghost membranes. The second peak was centered at f_γ1sp_, the characteristic frequency of γ1_sp_-relaxation (Figure 5B, open circles), and its negative direction conformed to the local increase on the ΔC″ vs. f curve. The result shown in Figure 5B is in line with the assumption that such a mild delipidation of erythrocyte ghost membranes did not remove the original closed lipid bilayer from the prepared Triton shells, although the content of this bilayer could be altered. This assumption is supported by the fact that (i) the capacitance contribution curve, ΔC′ vs. f, of Triton shells (Figure 5B, full circles) was comparable by amplitude with that of intact erythrocytes (Figure 5A, full circles); and (ii) the value of the characteristic frequency of interfacial β-polarization in these Triton shells was close to that in erythrocytes.

The two curves of complex capacitance contribution of moderately delipidated Triton shells (Figure 5C) demonstrate only the γ1_sp_-relaxation, while the β_sp_-relaxation was completely absent. Thus, the curves shown in Figure 5C for γ1_sp_-relaxation correspond to that part of Figure 5B which is surrounded by the dashed rectangle. This result is in accord with the curves presented on the ΔY″ vs. ΔY′ plots of such shells (Figure 3B,C) and suggests extensive removal of the original discontinuous lipid bilayer from the shells prepared at such concentrations of Triton-X-100. The absence of a closed lipid bilayer in these shells is underlined by the zero amplitude of their capacitance contribution curve, ΔC′ vs. f (Figure 5C, full circles). Because the curves of complex capacitance contribution (Figure 5C) were obtained with Triton shells packed in media with low (10 mM) concentration of NaCl, their characteristic frequency, f_γ1sp_, of γ1_sp_-relaxation had very low value (about 1.5 MHz), as is shown in Figure 4A. 

Considering the bell-shaped dielectric loss curve for erythrocytes (Figure 5A, open circles), let us suggest that there was no other relaxation in front of β_sp_-relaxation and behind it. Because the β_sp_-relaxation is a single-time relaxation, its dielectric loss curve should have a perfectly symmetric shape with respect to the vertical axis passing through its top point. In such a case, provided the left shoulder of this bell-shaped curve is rotated at 180° around this axis, it should coincide with the right shoulder. Figure 5A (the curve of grey circles) shows that it actually arrives at a position a little bit away from the right shoulder. The deficient grey area between the grey symbol curve and open symbol curve, which occupies the frequency range of γ1_sp_-relaxation, could be ascribed to the sought negative dielectric loss curve of γ1_sp_-relaxation. In subtracting the experimentally obtained open symbol curve from the grey symbol curve, one obtains the sought negative dielectric loss curve, ΔC_ds_″ vs. f, for the γ1_sp_-relaxation (not shown). Figure 5A shows that the middle- and high-frequency parts of the ΔC′ vs. f curve of γ1_sp_-relaxation (the bottom and ascending part of ΔC′ vs. f curve) were experimentally obtained. The remaining low-frequency part could be obtained by extrapolation as a plateau at lower frequencies (Figure 5A, grey circles). 

**Figure 6:**
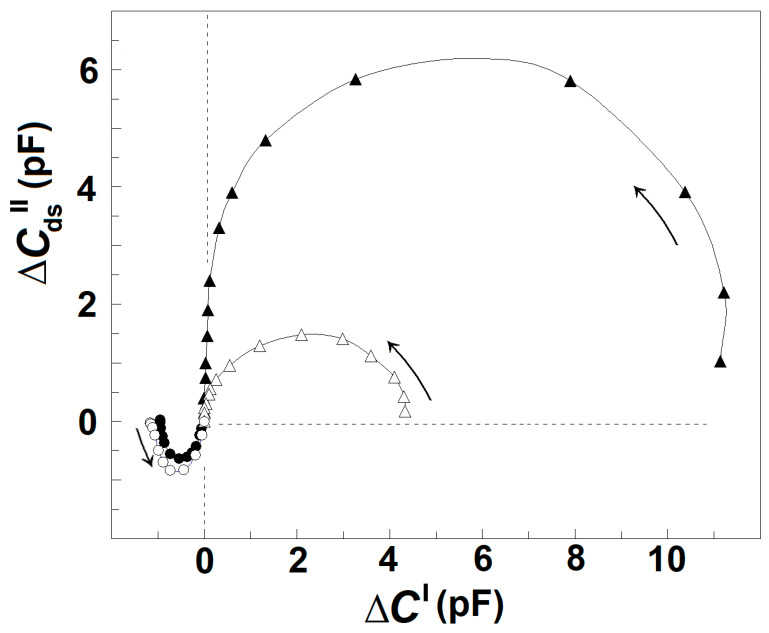
Reconstructed complex plain plots of capacitance contribution, ΔC_ds_″ vs. ΔC′ of the spectrin network of intact and acid-treated erythrocytes. The ΔC_ds_″/pF, corrected as described for the Figure 5A, is represented as function of corrected ΔC′/pF. The erythrocytes were treated in 130 mM NaCl saline, containing 25 mM citrate/HCl buffer with pH 7.4 (▲) or pH 5.2 (Δ), hematocrit 10%, at 4 °C for 90 min. The suspension of treated erythrocytes was centrifuged, and the isolated paste of packed cells tested.

Thus, the procedure described above allows us to obtain the reconstructed ΔC_ds_″ vs. f and ΔC′ vs. f curves for the γ1_sp_-relaxation. Applying this procedure for the intact erythrocytes and acid-treated erythrocytes, one obtains their reconstructed (actual) ΔC_ds_″ vs. ΔC′ plot, as shown in Figure 6. For each tested frequency, the reconstructed ΔC′ and ΔC_ds_″ values were both positive for β_sp_-relaxation and negative for γ1_sp_-relaxation, and they both tended to zero for f above 10 MHz. Compared to the reconstructed plot of intact erythrocytes, the reconstructed plot of acid-treated erythrocytes indicated the strength of β_sp_-relaxation about fourfold reduced; nevertheless, the strength of γ1_sp_-relaxation was quite the same for the two sample erythrocytes. This result indicates that the low-pH treatment (and possibly the limited extraction of lipids) of erythrocyte membrane specifically inhibited β_sp_-relaxation only.

### 3.3. Complex Admittance Contribution of Spectrin Network as Affected by the Inter-Membrane Interaction

#### 3.3.1. Effect of Extracellular and Intracellular NaCl on the Strengths of β_sp_- and γ1_sp_-Relaxations in Erythrocytes and Erythrocyte Ghost Membranes

**Figure 7:**
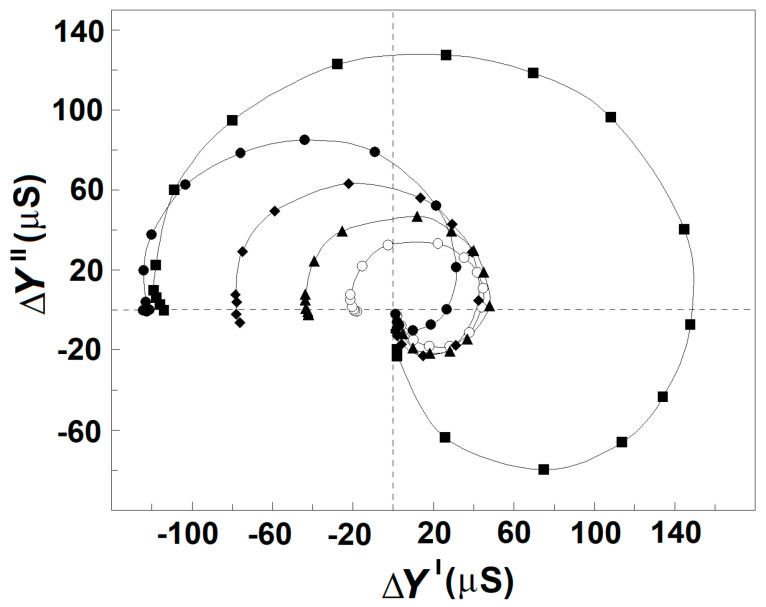
Effect of the concentration of NaCl and blood plasma in extracellular medium on the complex plain plot of admittance contribution, ΔY″ vs. ΔY′, of the spectrin network in erythrocytes. ΔY″/μS is presented as a function of ΔY′/μS. The erythrocytes were suspended at hematocrit of 45% in isotonic media containing 10 mM NaCl and 280 mM mannit (○), 50 mM NaCl and 200 mM mannit (▲), 100 mM NaCl and 100 mM mannit (♦), 150 mM NaCl (●), and homologous blood plasma thrice diluted by 150 mM NaCl (■). The model plots are omitted for clarity. Other details are presented in Figure 2A.

**Figure 8:**
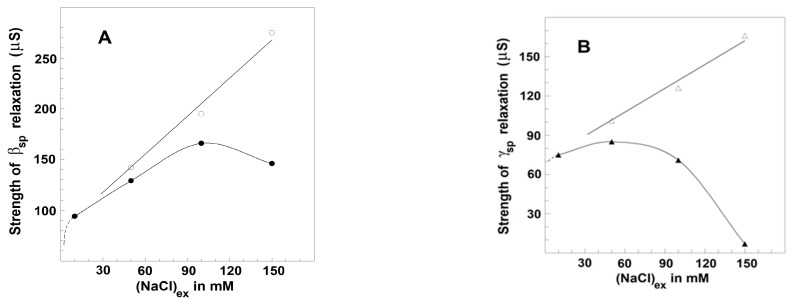
Effect of extracellular NaCl and blood plasma on the strengths of β_sp_-relaxation (**A**) and γ1_sp_-relaxation (**B**) in erythrocytes. The strength of β_sp_-relaxation, Y_βsp_ (μS) (●), and the strength of γ1_sp_-relaxation, -Y_γ1sp_ (μS) (▲), are presented as functions of extracellular NaCl concentration without (full symbols) and at the presence (open symbols) of three time diluted plasma. The erythrocytes were suspended at hematocrit of 45% in isotonic media containing mannit and the indicated concentration of NaCl with or without diluted plasma. Other details are presented in Figure 7.

Figure 7 exhibits how the strengths of β_sp_- and γ1_sp_-relaxations in erythrocytes varied when the concentration of NaCl in the suspension media was increased from 10 to 150 mM. The erythrocytes were suspended at the hematocrit of 45% in isotonic NaCl/mannit media. To make the suspension media isotonic at different NaCl concentrations, proper amounts of mannit were added. The latter was considered membrane impermeable and a non-reactive dielectrically passive ingredient not affecting the strength of relaxations. As the isotonic media with extremely low ionic content is known to compromise the permeability barrier of erythrocyte membranes [48], the concentrations of NaCl above 10 mM were only tested in this experiment. The increase in NaCl concentration from 10 to about 100 mM was accompanied by almost-linear enhancement of the strength, Y_βsp_, of β_sp_-relaxation, while the strength, Y_γ1sp_, of γ1_sp_-relaxation practically remained constant (Figure 7 and Figure 8, full symbols). A further increase in outside NaCl concentration from about 100 mM to 150 mM reversed the outlined tendencies, slightly decreasing the strength of β_sp_-relaxation and collapsing the strength of γ1_sp_-relaxation. 

As shown in Figure 7 and Figure 8 (full symbols) for erythrocytes, the outside NaCl produced a similar two-phase effect on the dielectric relaxations in erythrocyte ghost membranes. Such ghost membranes had cytosolic osmolarity and conductivity close to those of intact erythrocytes and were produced by resealing the membranes of hemolyzed erythrocytes in isotonic medium of 2 mM MgCl_2_, 5 mM phosphate buffer, pH 7.8, 75 mM NaCl, and 150 mM mannit.

In another set of experiments, erythrocyte ghost membranes were resealed with the media of 2 mM MgCl_2_, 5 mM phosphate buffer, pH 7.8, and NaCl at various concentrations. The ghost membranes were suspended in media containing proper concentrations of mannit to achieve isotonicity and NaCl at a concentration 10 times smaller than the concentration of inside-NaCl in order to minimize the effect of outside-NaCl with respect to that of inside-NaCl. Increasing the inside concentration of NaCl from 20 to 100 mM, the strength of β_sp_-relaxation linearly increased, while that of γ1_sp_-relaxation remained almost constant, similarly to that shown in Figure 7. Similar result was obtained with inside-NaCl concentration of 150 mM; however, the repeatability was poor. The repeatability strongly improved when the inner medium of NaCl contained 40 mM mannit (a substitute of hemoglobin?). In case the inside NaCl was replaced by KCl (which is the physiologically relevant component of cytosol), the above dependencies lasted until 150 mM KCl (not shown). The inner aspect of the lipid membrane is more complex compared to the outside one. Hence, some additional factors (trans-membrane voltage difference, water activity, the type of ions present) could also be involved that remain to be studied. 

#### 3.3.2. Effect of Extracellular Albumin on the Strengths of β_sp_- and γ1_sp_-Relaxations in Erythrocytes

To shed light on the above-mentioned two-phase effect of outside NaCl, part of the outside NaCl solution was replaced by homologous blood plasma (an isotonic solution of about 70 mg/mL proteins, predominantly albumin, 5 mM KCl, 5 mM CaCl_2_, and 130 mM NaCl). Figure 7 shows the effect on the strength of the dielectric relaxations in erythrocytes produced by the presence of blood plasma in the outside media. Prior to the suspension of erythrocytes, different volumes of the plasma were diluted by isotonic 150 mM NaCl solution; thus, the final concentrations of NaCl in the obtained suspension media were always close to 150 mM. When the proportion between the volumes of plasma and diluting NaCl solution was 1:5 and lower (the residual albumin concentration about 10 or less mg/mL), the two relaxations were as much inhibited as if the erythrocytes were suspended in 150 mM NaCl (not shown). With blood plasma diluted between four and two times (albumin concentrations between 15 and about 30 mg/mL), the two relaxations became strongly enhanced (Figure 7).

In the next experiment, homologous plasma was diluted by isotonic solutions containing proper combinations of NaCl and mannit in order to produce final isotonic suspension media with the desired concentration of albumin and various concentrations of NaCl. In cases where the suspension media contained homologous plasma diluted five or more than five times, the strengths of relaxations depended on the concentration of outside NaCl as if there was no plasma in the suspension media (Figure 8, full circles). In this case, the albumin, introduced by the diluted plasma in the suspension media, was close to the critical value of about 10 mg/mL or less. Such a concentration of albumin was far more than that (1–3 mg/mL) used to restore and maintain the discocytic shape of erythrocytes, which is in line with earlier claims [24] that the shape transformations at T_A_ were not important for the detected dielectric changes. 

In cases where the suspension media contained plasma diluted between four and two times, the strengths of β_sp_- and γ1_sp_-relaxations strongly and linearly increased when the NaCl concentration was increased from 10 to 150 mM (Figure 8, open circles). In the latter case, the plasma introduced albumin with a final concentration between 15 and 30 mg/mL. The enforcement of the two relaxations was apparent at any concentration of NaCl in outside media, especially at the reversal stage when NaCl concentration was between 100 and 150 mM (Figure 8, open circles). 

However, the plasma, diluted three times by 150 mM NaCl saline, its enforcing effect on the strengths of erythrocyte relaxations lost to a greater extent in cases where it was pre-heated (85 °C, 30 min) and cooled down (23 °C) prior to the addition of erythrocytes (not shown). This finding suggests that some plasma proteins, which became denatured during the pre-heating, were needed in order for the plasma to demonstrate its enhancement on the dielectric relaxations in erythrocytes. 

In the above experiment with erythrocytes suspended in the blood plasma diluted by NaCl saline, the suspension media contained KCl and CaCl_2_ at millimolar concentrations introduced by the plasma itself. The next experiments were designed to probe the effect of these ions on the strengths of β_sp_- and γ1_sp_-relaxations. When the erythrocytes were suspended in NaCl/mannit medium containing either 10 mM KCl or 5 mM CaCl_2_, no additional effects were detected, and the strengths of β_sp_- and γ1_sp_-relaxations depended only on the concentration of NaCl as it shown in Figure 7 and Figure 8 (full circles). 

The above results substantiate that the minor ion components of blood plasma, KCl and CaCl_2_, even at twofold higher concentration, were not important, and the detected effect on the strengths of dielectric relaxations was possibly due to plasma proteins, primarily albumin. In support of this suggestion, similar results to those obtained by the presence of homologous plasma were produced when the suspension media contained pure albumin isolated from bovine serum. In case the albumin concentration was less than the critical concentration of about 10 mg/mL, corresponding to about five times the dilution of the plasma, the strengths of β_sp_- and γ1_sp_-relaxations depended only on the concentration of NaCl, as is shown in Figure 7 and Figure 8 (full circles). However, when the albumin concentration was between 15 and 30 mg/mL, corresponding to plasma dilution between four and two times, the strengths of β_sp_- and γ1_sp_-relaxations were enhanced at any concentration of NaCl up to 150 mM (Figure 7 and Figure 8, open circles).

The two lines of experiments, taken together, lead to the following conclusion. The presence in extracellular media of either pure albumin or the albumin of blood plasma at concentrations above the critical one (about 10 mg/mL) was accompanied by huge enforcement of erythrocyte relaxations, elimination of the reversal downhill stage above 100 mM NaCl, and continuation of the initial ascending stage up to 150 mM NaCl. Thus, the augmentation effects on the relaxations in erythrocytes, produced by albumin and NaCl, were especially pronounced when the concentrations of both NaCl and albumin were close to their physiological levels.

#### 3.3.3. Effect of Extracellular Synthetic Polymers on the Strengths of β_sp_- and γ1_sp_-Relaxations in Erythrocytes

**Figure 9:**
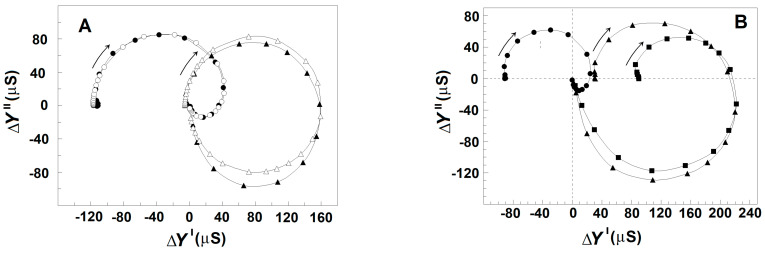
Effect of outside polymers on the complex plain plot of admittance contribution, ΔY″ vs. ΔY′ of the spectrin network in erythrocytes, suspended at hematocrit of 45%. ΔY″/μS is presented as a function of ΔY′/μS. (**A**) The erythrocytes were suspended in 150 mM NaCl (●) and in 150 mM NaCl containing 25 mg/mL polyvinylpyrrolidone (▲). The open symbols indicate model plots. (**B**) The erythrocytes were suspended in 150 mM NaCl (●) and in 150 mM NaCl containing polyethylene glycol at concentrations 20 mg/mL (▲) and 30 mg/mL (■). Other details are the same as for Figure 2A.

Results similar to those obtained with albumin were produced by polyvinylpyrrolidone and polyethylene glycol with molecular weights of 40 kDa and dextran with a molecular weight of 7 kDa. Like the natural polymer albumin, they are membrane-impermeable and membrane-inactive polymers. Dissolved in the suspension media at concentration above 10 mg/mL, they also tended to enhance the relaxations in erythrocytes suspended in 150 mM NaCl (Figure 9). However, they were not as perfect as albumin, which almost equally enhanced both β_sp_- and γ1_sp_-relaxations in erythrocytes (Figure 7). While the γ1_sp_-relaxation was strongly (about ten times) enhanced by the three synthetic polymers, the strength of β_sp_-relaxation was not changed in the presence of polyvinylpyrrolidone (Figure 9A) and was slightly enhanced in the presence of polyethylene glycol and dextran (Figure 9B).

#### 3.3.4. Effect of Cellular Packing on the Strength of Relaxations in Erythrocytes

With erythrocytes suspended in isotonic medium of 10 mM NaCl and 280 mM mannit, increasing the hematocrit value has been shown to enhance the strength of detected relaxations almost linearly up to the hematocrit of about 45%, and then to the hematocrit of about 90% with apparent saturation [25]. This indicates the weak impact of intermembrane interaction, which slightly inhibited the relaxations only at the substantial nearness of cells about the hematocrit of 45%. At such hematocrit values (40–90%), a variation in hematocrit of about ±2 to 3% did not produced detectable deviation in the strength of relaxations. However, when the packing of erythrocytes were close to the maximal one (obtained via extensive centrifugation), the strength of detected relaxations became markedly inhibited. 

**Figure 10:**
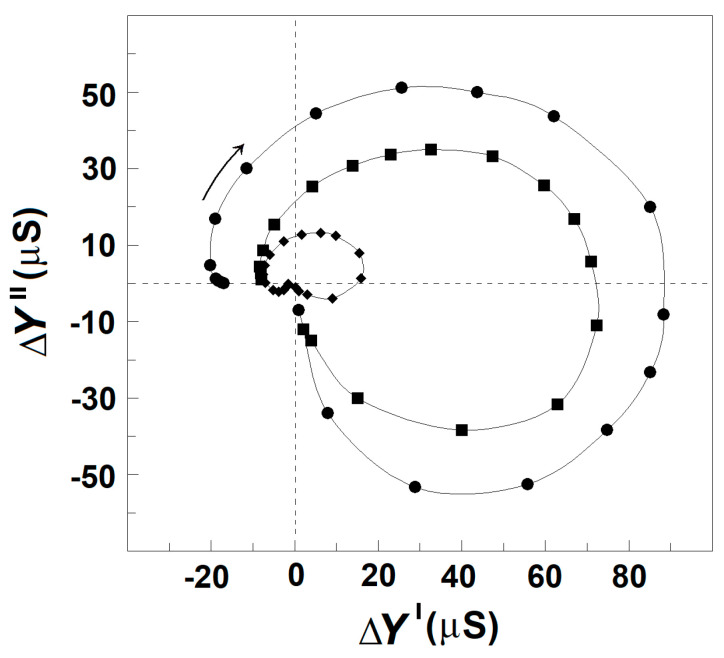
Effect of cellular packing on the strength of dielectric relaxations in erythrocytes. Δ*Y*″/μS is presented as function of Δ*Y*′/μS. The erythrocytes were suspended in isotonic medium of 10 mM NaCl saline and 280 mM mannit and packed to the maximal hematocrit at the following conditions: centrifugation at 150× *g* for 10 min (●), centrifugation at 4000× *g* for 20 min (♦), and centrifugation at 4000× *g* for 20 min in the indicated medium to which 30 mg/mL albumin was added (■). The model fits are not shown for clarity. Other details are the same as for Figure 2A.

To demonstrate this effect, the tested erythrocytes were suspended at the hematocrit of about 80% in the isotonic medium of 10 mM NaCl and 280 mM mannit with or without 30 mg/mL albumin. The obtained suspension was centrifuged to prepare two types of pellets of packed erythrocytes. The pellets of loosely packed erythrocytes were prepared by mild centrifugation (150× *g*, 10 min) and had the hematocrit of about 94%. The pellets of densely packed erythrocytes were prepared by harsh centrifugation (4000× *g*, 20 min) and had the hematocrit of about 97% [49]. Both types of pellets were sufficiently leaky, allowing 70 μL of each pellet to be aspirated by a syringe through a plastic tube with 1 mm inner diameter and inserted into the conductometric cuvette to detect the β_sp_- and γ1_sp_-relaxations using the ΔY″ vs. ΔY′ plot (Figure 10). The plot of pellets containing erythrocytes, loosely packed without albumin (Figure 10), did not differ substantially from the plot of erythrocytes suspended at hematocrit of 80% in the isotonic medium of 10 mM NaCl and mannit (not shown). This indicates that the additional act of bringing the cells closer to each other, due to the applied mild centrifugation, did not produce inhibition of the relaxations in excess of that present in the suspension with 80% hematocrit. However, compared to the pellets of erythrocytes loosely packed without albumin, the strength of relaxations was inhibited by about 500% in the pellets of erythrocytes densely packed without albumin, and only by about 40% in the pellets of erythrocytes densely packed at the presence of albumin (Figure 10). These results substantiate that severe cellular packing markedly inhibited the strength of dielectric relaxations in erythrocytes, and this inhibition was by far obviated by albumin present in the suspension media at overcritical concentrations.

## 4. Discussion

### 4.1. Role of Lipid Membrane in the Dielectric Relaxations on the Spectrin Network

The increase up to about 100 mM in the concentration of NaCl either in the outside or inside media, at preserved isotonicity, allowed a greater charge to be accumulated through the Maxwell–Vagner effect on the lipid bilayer of erythrocytes and erythrocyte ghost membranes. In addition, the strength of β_sp_-relaxation increased linearly with the concentration of NaCl, while the strength of γ1_sp_-relaxation remained constant (Figure 3A,B). Second, upon the gradual extraction of erythrocyte membrane lipids, especially the part of the lipid bilayer which includes the glycophorin C integral protein, the β_sp_-relaxation was increasingly inhibited, while the strength of γ1_sp_-relaxation was preserved (Figure 4). Third, the detachment of erythrocyte lipid membrane from the spectrin network via severing the bridge between the glycophorin C and actin–spectrin junction at pH 5.2 was accompanied by substantial inhibition of β_sp_-relaxation and preservation of the strength of γ1_sp_-relaxation (Figure 6).

The above results possibly indicate that glycophorin C, intercalated in the lipid bilayer of erythrocytes, together with the lipid bilayer itself, were both needed for the strength of β_sp_-relaxation. On the other hand, they both appeared not to be needed for the γ1_sp_-relaxation, since the overcritical delipidation of erythrocyte ghost membranes preserved this relaxation and completely removed the β_sp_-relaxation. The preservation of γ1_sp_-relaxation in slightly and moderately delipidated shells implies that the lipid bilayer served rather as an obstacle for the field to enter the cytosol and interact with the dipoles of spectrin. The above results provide additional support to the already proposed mechanisms explaining the two relaxations. 

### 4.2. Biophysical Characteristics of β_sp_-Relaxation

The characteristic frequency of β_sp_-relaxation was strictly coupled to the characteristic frequency of the interfacial β-relaxation on the lipid bilayer of erythrocyte plasma membrane. As the frequency range of the β_sp_-relaxation is low, at least its initial part, this relaxation has been assumed based on the indirect interaction of the incident electric field with the spectrin filaments [1,24]. The results presented in this study indicate that the electric field-driven alternating accumulation of charges on either side of the lipid bilayer could be the energy source of this relaxation. The electrostriction of the lipid bilayer, consequent to charge accumulation, produced transversal mechanical oscillations which were conveyed through the attachment sites, mainly through the attachment of glycophorin C integral protein to the actin–spectrin junction. The extreme rigidity of actin oligomer and its connectivity with an average five to seven spectrin filaments ensures an efficient transmission of this vibrational energy to spectrin filaments whose transversal oscillations, in turn, generated piezo electricity.

This conception assumes that intact spectrin was only able to exhibit the piezo effect, and this ability was completely lost after the rapid unfolding of spectrin at T_A_. It stays in line with the result that the dielectric energy loss, ΔC_ds_″(f) (Figure 5A), spent on the spectrin network and the contribution of the spectrin network to the capacitance, ΔC′(f) (Figure 5A), of the erythrocyte plasma membrane both demonstrated positive signs over the frequencies of β_sp_-relaxation. In addition, specific permeabilization of erythrocyte lipid membranes by saponin, as reported recently [2], reduced the charge accumulation on the lipid bilayer and inhibited the ΔC_ds_″(f) and ΔC′(f) curves associated to the β_sp_-relaxation. 

### 4.3. Biophysical Characteristics of γ1_sp_-Relaxation

Over the frequency range of γ1_sp_-relaxation, the alternating field freely enters the erythrocyte cytosol. Hence, the field has been assumed to come into resonance with the natural (thermal) oscillations of dipoles of spectrin segments [2], whose amplitude could hardly depend on the outside concentration of NaCl. As the absorbed electric energy during the γ1_sp_-relaxation will depend on the amplitude of these oscillations, it is not expected to depend on the outside concentrations of NaCl in line with the results presented in Figure 8A,B.

Compared to the ΔC″ vs. ΔC′ plot, the ΔY″ vs. ΔY′ plot appears more suitable for comparative study of the two dielectric relaxations in erythrocytes as the complex admittance contribution of the spectrin network during the γ1_sp_-relaxation was sufficiently large, with both positive real part and negative imaginary part (Figure 2 and Figure 6). By contrast, the respective complex capacitance contribution during the γ1_sp_-relaxation was quite faint, and only its negative real part could be registered as a small drop in the ΔC′ vs. f curve at f_γ1sp_ (Figure 5A, full circles). Thus, a question arises: is there dielectric loss at all during the γ1_sp_-relaxation, and, furthermore, what might be its sign?

In a recent study, this problem was resolved using the acidification of erythrocytes and the limited extraction of erythrocyte ghost membranes by Triton-X-100. Both means inhibited β_sp_-relaxation and apparently enhanced γ1_sp_-relaxation, obtaining reliable-by-amplitude complex capacitance contribution of the spectrin network during the γ1_sp_-relaxation, whose real and imaginary parts both had negative signs (Figure 5B,C). Hence, it was suggested that the dielectric loss curve of the spectrin network in erythrocytes during γ1_sp_-relaxation was too low by amplitude and superimposed by the nearby powerful β_sp_-relaxation. The procedure described in Figure 5A for obtaining the reconstructed (actual) curves of ΔC′ vs. f and ΔC_ds_″ vs. f for γ1_sp_-relaxation represents an attempt to better separate the two relaxations on the frequency axis. Applying this procedure for the intact erythrocytes, one obtains their actual ΔC_ds_″ vs. ΔC′ plot (Figure 6), which indicates substantial-by-amplitude and negative-by-sign dielectric loss during the γ1_sp_-relaxation. This outcome could be supported by the following considerations. 

First, from the equation Y* = Y′ + jY″, where Y′ = ωC_ds_″ and Y″ = ωC′ [50], it follows that

Δ*C*′ = Δ*Y*″/*ω* (1)

Δ*C*_ds_″ = Δ*Y*′/*ω*. (2)

Hence, compared to the ΔY″ vs. ΔY′ plot, the ΔC_ds_″ vs. ΔC′ plot will exhibit the same β_sp_- and γ1_sp_-relaxations with the same signs. However, due to the reciprocal dependence on the circular frequency, ω, the high-frequency (γ1_sp_) relaxation will be expressed on the ΔC_ds_″ vs. ΔC′ plot, compared to the same relaxation on the ΔY″ vs. ΔY′ plot with about seven-times-smaller strength, as f_γ1sp_/f_βsp_ = 9 MHz/1.3 MHz = 6.92. Indeed, compared to the semicircle of β_sp_-relaxation, the semicircle of γ1_sp_-relaxation attached at the left (high frequency) end of the reconstructed ΔC_ds_″ vs. ΔC′ plot of intact erythrocytes (Figure 6) was about tenfold reduced, which is in full compliance with the above conclusion drawn from the Equations (1) and (2).

According to recent reports [1,2], γ1_sp_-relaxation in erythrocytes has been inhibited by a number of agents and conditions (treatment of erythrocytes by 5 mM N-ethylmaleimide and 5 mM ortho-vanadate, incubation of erythrocytes in medium with pH 9.2, and shrinkage of erythrocytes in hypertonic media (<700 mOsm) and in isotonic media using various ionophores), all known to detach significant number of band-3 ankyrin bridges from their association to the network of spectrin tetramers. On the other hand, the specific disconnection of the band-3 ankyrin bridge from the spectrin network (for example, by the treatment of erythrocytes with 5 mM N-ethylmaleimide) has been shown to dissociate a large part of spectrin tetramers into dimers on the erythrocyte membrane [3]. These results imply that spectrin tetramers were the dielectrically active participants in γ1_sp_-relaxation in erythrocytes, and their preliminary dissociation into dimers removed their contribution to the γ1_sp_-relaxation. This conclusion could be used to assess the dimer/tetramer ratio in a given erythrocyte sample.

### 4.4. Dielectric Relaxations in Erythrocytes as Sensitive Markers of Erythrocyte Membrane Deformability 

Recent reports have indicated that the strengths of the two relaxations were not affected by agents (4,4′-diiso-thiocyanato stilbene-2,2′-disulfonic acid, dithiothreitol, concanavalin A) known to modify the lipid membrane without affecting the spectrin network and deformability of erythrocytes [2,20]. By contrast, they were strongly reduced by agents (diamide, taurine mustard, glutaraldehyde, hypertonic and hypotonic media, wheat germ agglutinin, N-ethylmaleimide, phenylhydrazine) that modify the spectrin network and reduce the ability of erythrocyte membrane to deform and exhibit vibratory motions, referred to as “flickering″ [2,20]. Of these agents, the impairment of erythrocyte deformability produced by hypertonic media and N-ethylmaleimide quantitatively correlated to the extent of the inhibition of β_sp_-relaxation [2,20]. This observation suggests that compared to γ1_sp_-relaxation, the β_sp_-relaxation could be a more relevant marker to express the ability of erythrocyte membrane to deform [2,20]. The results presented in this study bring additional data in support of this assertion. 

According to an earlier report [51], the incubation of erythrocytes in acidic medium with pH 5.2 at conditions similar to those applied in this study strongly reduces the deformability of erythrocyte plasma membranes. The results obtained in this study show that compared to intact erythrocytes, the strength of γ1_sp_-relaxation in acid-treated erythrocytes was roughly unchanged (Figure 6) or even slightly increased (Figure 2), while the strength of β_sp_-relaxation was strongly reduced (Table 1), correlating to the reduction of deformability. Thus, based on our previous reports [2,20], the inhibition of β_sp_-relaxation by acid-treatment of erythrocytes could be interpreted as a specific marker for impaired deformability of acid-treated erythrocytes. 

Polyvinylpyrrolidone at concentrations up to 20 mg/mL has been shown not to alter the deformability of erythrocytes [52]. This outcome is routinely used in modern techniques of ektacytometry to evaluate the deformability of erythrocytes suspended in the highly viscous solution of polyvinylpyrrolidone, which is assumed not to affect the erythrocyte deformability itself. In this study, polyvinylpyrrolidone (20 mg/mL) dissolved in the suspension medium (150 mM NaCl) of native erythrocytes led to strong (an order of magnitude) enhancement of γ1_sp_-relaxation, while the strength of β_sp_-relaxation remained preserved (Figure 9A). This result again showed that the change in the strength of γ1_sp_-relaxation strongly diverged from, while the change in the strength of β_sp_-relaxation closely pertained to, the alteration of erythrocyte deformability. 

The above results indicate that in contrast to γ1_sp_-relaxation, the inhibition of β_sp_-relaxation could be interpreted, based on our previous reports [2,20], as specific marker for impaired deformability of erythrocytes. However, some agents (diamide, glutaraldehyde, wheat germ agglutinin, taurine mustard, phenylhydrazine) were not able to discriminate, quantitatively, some of the two relaxations as possibly indicative for impaired erythrocyte deformability. The reason was that fibrillar spectrin is an obligatory participant in both the β_sp_- and γ1_sp_-relaxations and each agent which stiffens spectrin; thereby, the erythrocyte membrane should inhibit nonspecifically the two relaxations. By contrast, acid-treatment which preserves both the spectrin and γ1_sp_-relaxation should exhibit how the glycophorin C disconnection from spectrin affects the deformability and β_sp_-relaxation. 

Based on the above indicated relationship of β_sp_-relaxation to the erythrocyte membrane deformability, and taking into account the molecular mechanism of β_sp_-relaxation, a novel hypothesis is here put forward. Briefly, the transformation of mechanical energy of erythrocyte membrane deformation into electric energy of the spectrin network could be a reversible one and is implicated as an adjuvant mechanism in the elasticity and flicker of erythrocytes plasma membrane. Briefly, similar to the electrostriction of the lipid membrane, each deformation of erythrocyte membrane produced during the blood circulation and by the flicker of erythrocytes will power up the direct piezo effect on the spectrin network, causing its dielectric polarization. After the removal of the deformational force, the electric energy, accumulated as dielectric polarization on the spectrin filaments, could be transformed through the reversed piezo effect back into mechanical force, helping restore the initial shape of the deformed membrane. Such a mechanistic view about the involvement of the spectrin network in the elasticity and flicker of the erythrocyte membrane is supported by recent findings that the strength of β_sp_-relaxation senses the artificially induced changes in the deformability and flicker of erythrocyte plasma membrane [2,20].

### 4.5. Dielectric Relaxations in Erythrocytes as Sensitive Markers of Inter-Membrane Interaction

In erythrocyte suspensions, closing the gap between erythrocytes and consequent aggregation is prevented by the electrostatic repulsion between the counter ion layers of erythrocyte double electric layers [53]. At higher concentrations of ions in the suspension medium, the width of the counter ion layer becomes smaller than 1 nm, allowing cells to come frequently within a short distance of each other, especially in dense suspensions. We assume that these contacts inhibit the β_sp_- and γ1_sp_-relaxations in erythrocytes, which explains the weakening of these relaxations at outside-NaCl concentration between 100 and 150 mM (Figure 8). Such an explanation is supported by the result obtained with extremely dense suspensions, subjected to additional packing by centrifugation; the mild packing of erythrocytes did not impose extra inhibition of the relaxations, while the harsh packing did (Figure 10). Another result in support was provided by the presence in outside medium of inert polymers, both natural (albumin) and synthetic ones (polyvinylpyrrolidone, polyethylene glycol, dextran). While the polymers had no effect at concentrations less than about 10 mg/mL, above this (apparently critical) concentration, they prevented the inhibition of relaxations at high-ionic-strength media and even provided extra enhancement to the relaxations (Figure 7, Figure 8 and Figure 9). Concerning this effect of polymers, albumin appeared best suited as it rendered equal enforcement to both relaxations and even almost entirely obviated their inhibition in erythrocytes subjected to harsh packing (Figure 10). 

The potency of these polymers to prevent the contacts between erythrocytes at short distance has been extensively demonstrated by their ability to reduce the erythrocyte aggregation and mechanical damage of erythrocytes. The dimension of the hydrodynamic radius of a polymer or macromolecule is the main criterion of its erythrocyte aggregation capacity; if it does not exceed 4 nm, it prevents aggregation, and if it is more than 4 nm, it promotes aggregation [54]. Thus, in accordance with these data, molecular albumin (as well as the tested polyvinylpyrrolidone and polyethylene glycol with molecular weights of 40 kDa and dextran with molecular weight of 7 kDa) is a promoter of erythrocyte disaggregation. Compared to phosphate buffered saline (150 mM NaCl), the blood plasma, albumin, and polyethylene glycol with a molecular weight of 20 kDa have been shown to shield the erythrocytes in suspension, reducing erythrocyte aggregation and protecting erythrocytes from mechanical damage and related hemolysis [55,56]. Being an anionic protein, albumin could directly disaggregate negatively charged erythrocytes and reduce erythrocyte aggregation in blood [57]. By contrast, dextran with molecular weight of 500 kDa was a promoter of erythrocyte aggregation and, at concentration above 10 mg/mL, it strongly reduced both the β_sp_- and γ1_sp_-relaxations (not shown).

Consider an electric field within the frequency range where Maxwell–Wagner interfacial polarization occurs being applied to suspended erythrocytes from left to right. Due to the accumulation of cations on the left side and anions on the right side of each erythrocyte, an ionic dipole sets up, and it is pointed in the direction opposite to the field [58]. In case the field is inhomogeneous and has an intensity of hundreds and thousands of volts per meter, the interaction of the induced dipoles with the field gives rise to ponderomotive responses such as electrophoresis, dielectrophoresis, electrorotation, and electrodeformation of cells. In all these events, the accompanying intramembrane particle polarization (electric field-induced displacement of intramembrane proteins and lipids) is considered to be unimportant in comparison to the polarization of ions in neighbor media. When the field is a near homogeneous one, and hundreds and thousands of times weaker, as in this study, the above ponderomotive responses would not take place; however, another problem arises in dense suspensions. The induced ion dipole distorts the field around each erythrocyte and impinges on the ionic dipoles of neighbor cells [59,60] causing substantial deviation in the calculated electric parameters of suspended cells [61]. Hence, what appears important in the β_sp_- and γ1_sp_-relaxations in erythrocytes is the polarization (alternating displacement, movement, oscillation) of intramembrane dielectrically active particles. The results obtained in this study shed light on what type of intramembrane particles, in addition to spectrin, could participate in the β_sp_- and γ1_sp_-relaxations.

For β_sp_-relaxation, the above conclusion appears obvious in light of the indicated participation of the lipid bilayer and glycophorin C. The inhibition of β_sp_-relaxation during the close contacts between neighbor cells and the prevention of this inhibition by outside polymers stays in concert with the assumed participation in β_sp_-relaxation of glycophorin C, which could be affected by the outside inhibitory stimulus. Compared to β_sp_-relaxation, the γ1_sp_-relaxation demonstrated much more strongly pronounced sensitivity to inhibition during the close contacts between neighbor cells, which indicates the involvement of another transmembrane protein coupled to spectrin. Previous reports that disconnection of band-3 integral protein from its linkage to spectrin inhibits the γ1_sp_-relaxation points to band 3 as the participant which connects the spectrin network to outside media and conveys the outside inhibitory stimulus for the γ1_sp_-relaxation. 

A similar transmembrane effect on the deformability of erythrocyte membrane has been proposed for glycophorin A, a minor integral protein of erythrocyte membrane which specifically binds the lectin, called wheat germ agglutinin. In the non-liganded state, the cytoplasmic domain of glycophorin A is assumed to be free floating and not associated with the spectrin network. Ligand binding induces a conformational change which causes the cytoplasmic domain of glycophorin A to come into close contact with the spectrin network, resulting in decreased flexibility of the spectrin network, increased rigidity of erythrocyte plasma membrane [62], and inhibition of β_sp_- and γ1_sp_-relaxations [20]. 

Thus, to accommodate all experimental results, both published ones and those here presented, one could regard the glycophorin C and band 3 as possible participants in the electric field–induced vibrations of spectrin tetramer during β_sp_- and γ1_sp_-relaxations, respectively. Consequently, the two relaxations on spectrin should differ by the nature and place of their energy sources. At β_sp_-relaxation, the vibrations are assumed to be generated within the lipid bilayer and are conveyed to spectrin mainly via the glycophorin C-actin linkage. At γ1_sp_-relaxation, the vibrations are generated at the middle of the spectrin tetramer and are transferred to outside medium by the band 3 tetramer through the lipid bilayer, which is facilitated by the low bending rigidity of the lipid membrane alone [63]. 

The glycophorin C and band 3 are both glycoproteins possessing huge oligosaccharide moieties that project from the outside aspect of erythrocytes. Blocking the two integral proteins from outside should quench their electric field-induced vibrations and inhibit the two relaxations on spectrin. Relatedly, the oligosaccharide moieties of the most numerous membrane proteins—including glycophorin C and, especially, the most complex band 3—were likely involved in the inter-membrane contacts inhibiting the two relaxations. Based on this conception, to explain the inhibition of the two relaxations in an erythrocyte by the neighbor erythrocytes, we could resort to a bulk of published and unpublished results from our work indicating such inhibition to be a consequence of erythrocyte aggregation due to a variety of aggregation stimuli (wheat germ agglutinin [20] and other lectins, Zn^2+^, agglutinins, Alcian blue die, antibodies, deposition of plasma immunoglobulins on the erythrocyte surface). Thus, the β_sp_-relaxation appears most sensitive to the deformability and elasticity of erythrocytes, γ1_sp_-relaxation could be used as a potential means to study erythrocyte aggregability; both problems represent major challenges to modern medicine. 

## Figures and Tables

**Table 1:** Effect of acidic pH on the model parameters of *β_sp_* and *γ1_sp_* dielectric relaxations on the spectrin network of erythrocytes. The erythrocytes were subjected to modification in media with the indicated pH. Other details are contained in Figure 2A. This and the next tables represent the typical result out of at least three experiments.

	−Y_βsp_, (μS)	−C_βsp_, (pF)	Y_γ1sp_, (μS)	C_γ1sp_, (pF)	−Y_βsp_/Y_γ1sp_	−C_βsp_/C_γ1sp_
pH 7.4	90.50	12.01	65.00	1.59	1.39	7.52
pH 5.2	57.00	6.98	77.00	2.04	0.74	3.41

**Table 2:** Effect of the low and moderate delipidation of erythrocyte ghost membranes on the model parameters of β_sp_- and γ1_sp_-dielectric relaxations in Triton shells. The erythrocyte ghost membranes were subjected to delipidation by Triton-X-100 at the indicated concentrations. Other details can be found in Figure 3.

	Concentration of Triton-X-100 (%Vol.)	−Y_βsp_, (μS)	−C_βsp_, (pF)	Y_γ1sp_, (μS)	C_γ1sp_, (pF)	−Y_βsp_/Y_γ1sp_	−C_βsp_/C_γ1sp_
Erythrocyte ghost membranes	-	103.00	12.62	85.00	1.50	1.21	8.40
Triton shells (low delipidated)	0.07	105	10.45	184.7	2.45	0.57	4.29
Triton shells (moderate delipidated)	0.10–0.20	5.0	0.72	110	2.2	22	3.025

## Abbreviations

Triton-X-100, 2-[4-(2,4,4-trimethylpentan-2-yl)phenoxy]ethanol; spectrin network, spectrin-based submembrane skeleton; *T*_A_, temperature for denaturation of spectrin; *f*, frequency of electric field; *f*_βsp_, characteristic frequency of *β_sp_*-relaxation on spectrin network; *f*_γ1sp_, characteristic frequency of *γ1_sp_*-relaxation on spectrin network.
